# Comparison of proprioceptive acuity of the cervical spine in healthy adults and adults with chronic non-specific low back pain: A cross-sectional study

**DOI:** 10.1371/journal.pone.0209818

**Published:** 2019-01-10

**Authors:** Maria Emmert, Corina Schuster-Amft, Eling D. de Bruin, Michael A. McCaskey

**Affiliations:** 1 Research Department, Reha Rheinfelden, Rheinfelden, Switzerland; 2 ZHAW School of Health Professions, Winterthur, Switzerland; 3 Institute of Rehabilitation and Performance Technology, Bern University of Applied Sciences, Burgdorf, Switzerland; 4 Department of Health Sciences and Technology, ETH Zurich, Zurich, Switzerland; 5 Division of Physiotherapy, Department of Neurobiology, Care Sciences and Society, Karolinska Institutet, Stockholm, Sweden; Universitat de les Illes Balears, SPAIN

## Abstract

**Background:**

It has been suggested that patients with chronic non-specific low back pain (CNSLBP) perform poorly in postural tasks when compared to healthy individuals. Despite its importance in posture and alignment of the trunk in relation to the head, neck proprioception has not been examined in patients with low back pain. The purpose of this study was to compare neck proprioception in patients with CNSLBP with healthy individuals.

**Methods:**

Cervical joint reposition error was measured five times consecutively in the neutral head position, 30° and 60° left and right head rotation. The main outcome measure was the mean cervical joint repositioning error of the head.

**Results:**

Forty-six participants with (n = 24, 54 ± 16yrs SD, 14 females) and without (n = 22, 36 ± 13yrs SD, 13 females) CNSLBP were included in the study. Comparison of mean cervical joint repositioning error between patients and healthy controls showed no statistically significant group difference in any of the applied positions. The range of deviation in CNSLBP patients was between 1.57° and 3.27° compared to 1.46° to 2.26° in healthy controls. An overshooting tendency for both groups was found in the neutral head position.

**Conclusion:**

The ability to accurately position the head does not seem to be impaired in patients with CNSLBP. This may suggest that sensorimotor control is affected on other levels of the movement system and future research should focus on methods to identify the source of these aberrations.

## Introduction

Low back pain ranks first for years lived with disability and sixth for overall burden in the Global Burden of Disease study 2010 [1, 2]. The lifetime prevalence of low back pain in Europe has been estimated at 84% [3] and, according to the Federal Statistical Office of Switzerland, almost a quarter of patients treated for musculoskeletal diseases in hospitals suffered from back related disorders [4]. In low back pain, the relapse-rate of pain is about 44–78% and of work absence about 26–37%. Understanding its mechanisms and improving treatment remains a priority in the global attempt to improve quality of life and reduce health care costs [5].

Chronic, non-specific low back pain (CNSLBP) is defined as pain persisting for at least 12 weeks, localized below the costal margin and above the inferior gluteal folds, without known specific pathology such as radicular or cauda equina syndrome [3, 6, 7]. Low back pain is not attributable to specific pathology in more than 85% of cases [3, 7, 8]. While mechanisms leading to chronicity still remain poorly understood, risk factors suspected to contribute are individual (genetics, gender, age, body build, strength, and flexibility) and activity-related (work and leisure) factors [9]. Furthermore, the Clinical Guidelines of the American Physical Therapy Association suggest that psychosocial factors appear to play a prognostic role in CNSLBP [9].

Although no conclusive evidence of a causal relationship exists, it is generally accepted that posture plays an important role in the development of CNSLBP [7, 10, 11]. People with low back pain seem to adopt a rigid postural control strategy because of pain and fear of pain [7]. It is assumed that this leads to a vicious cycle of decreased movement, connective tissue remodeling, inflammation, nervous system sensitization and further decreased mobility, which leads to abnormal joint and tissue loading during daily activities. It has been suggested in previous studies, that this, in turn, may affect local proprioceptors [12–14]. Proprioception provides somatosensory input for the continuous control and correction of body position and orientation and its importance in postural control is undisputed [15]. Particularly neck proprioceptors play a central role in spatial orientation of the head with respect to the trunk, and in controlling posture against gravity. In their review, Pettorossi et al. [12] concluded that proprioception from the neck muscles contributed to the construction of cognitive representation of the body. This includes the position of limb segments, their hierarchical arrangement, and configuration of the segments in space [12]. However, as shown by a recent systematic review on proprioceptive interventions in chronic low back and neck pain, its role in musculoskeletal disorders is still unclear [16].

An important component of proprioception is the sense of position, which can be assessed in research and clinic using measurements of joint repositioning error (JRE) [10, 17, 18]. The sense of position is predominantly provided by the afferent input of muscle spindles, supposedly with the addition of cutaneous and joint receptors [10, 19]. Compared to other spinal regions neck muscles have the highest density of spindles [10]. Bearing in mind that the indirect measure of proprioception limits the interpretation regarding increased afferent input, several approaches suggest functional assessments of cervical proprioception by testing JRE: the ability to actively relocate the head to the neutral position or to a reference position [10, 12, 19–26].

Numerous studies have shown that compared to a pain-free population, people with low back or neck pain have altered lumbosacral [14, 27–34], or cervical JRE [20, 23, 24, 35–38], respectively. The results of the above mentioned studies indicate that JRE of the neck is generally higher in patients with neck pain compared to healthy controls. Despite its supposed importance in posture and alignment of the trunk in relation to the head, we are not aware of any studies investigating the relationship of neck proprioception and CNSLBP. Sensory deficits in patients with CNSLBP would point to the necessity to develop and employ methods that improve sensorimotor control. If specific components of proprioception are impaired in CNSLBP, then it will subsequently be important to know whether they are a cause or consequence of the pain and whether they can predispose to chronicity. Such findings would provide merit for studies which aim to improve proprioceptive and postural training to prevent chronicity in people with CNSLBP. Hence, the purpose of this study was to compare neck proprioception in patients with CNSLBP and in healthy individuals by measuring the cervical JRE. We hypothesized differences between CNSLBP patients and healthy controls in measures of neck proprioception.

## Materials and methods

### Study design

Measuring the JRE, this study applies a cross-sectional design to assess proprioception of the cervical spine in healthy adults compared to patients with CNSLBP. The data in the current study was collected from January until December 2015. The STROBE Statement guidelines were used to report the results of this cross sectional study [39]. The study was implemented as part of an approved and registered trial (ClinicalTrials.gov Identifier: NCT02304120). The individual depicted in this manuscript has given written informed consent (as outlined in PLOS consent form) to publish these case details.

### Setting and participants

**Fig 1:**
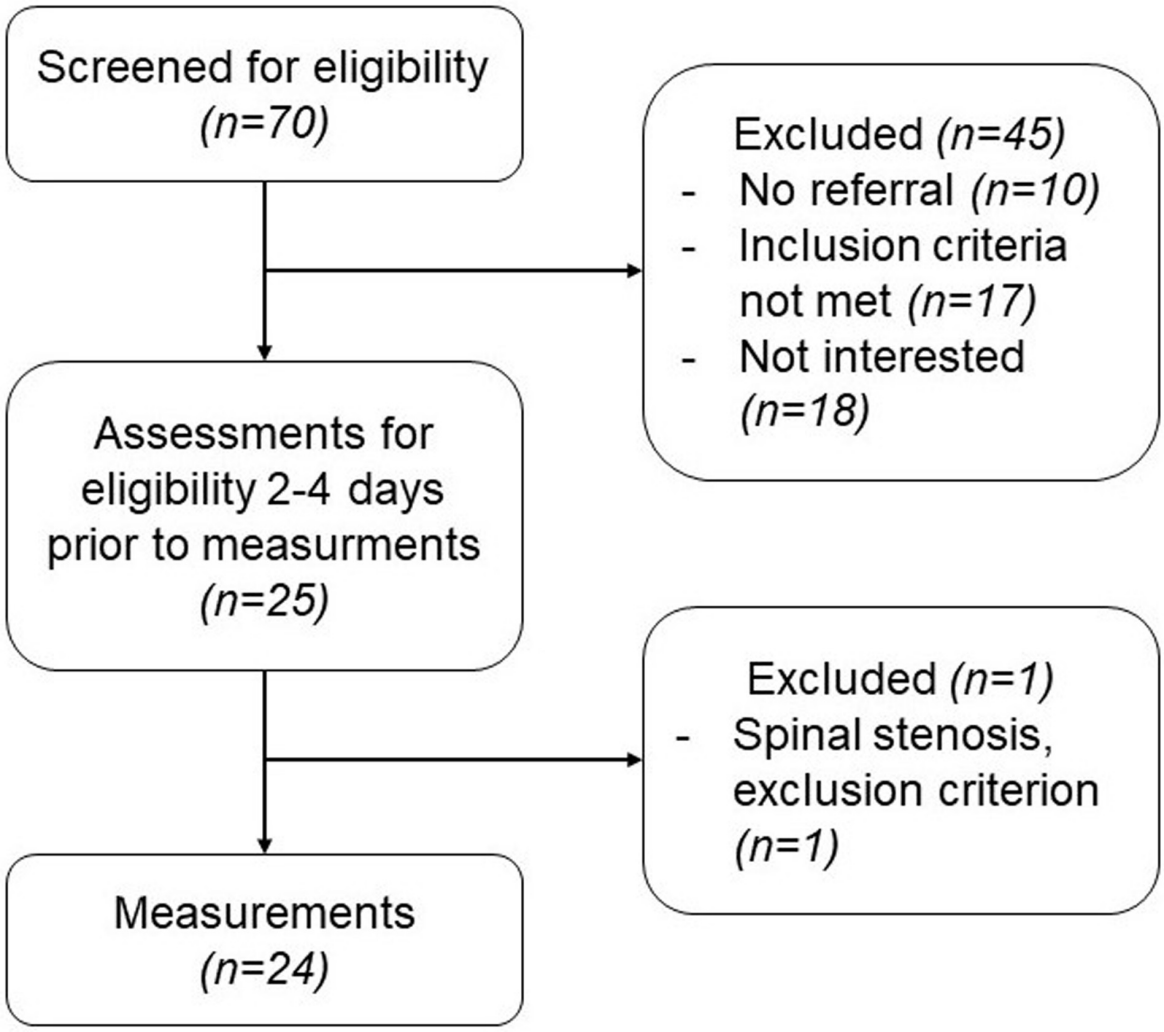
Flow-chart of CNSLBP patients’ recruitment. Process from initial contact with possible participants until participation in the study.

The study was conducted in a neuro-orthopedic rehabilitation center in Switzerland [40]. Participants were recruited after screening the patient admission lists of the study site. Additionally, participants were invited to participate through advertisement in local media, and the clinic’s homepage; local care providers and physicians were contacted for referrals. Detailed recruitment procedure can be seen in Fig 1.

An a priori power calculation was conducted (G*Power 3.1) [41], based on results of Loudon et al. [42] reporting significant differences in JRE of 30° left cervical rotation between whiplash and healthy controls. Weighted mean effect size was calculated by taking the means of the standard deviation of the two groups dividing it by the standard deviation of the whiplash group (r = 0.82). A sample size of 20 participants per group was determined in order to reach a power of 0.80 at an α-error of 0.05. Potential participants had contact with the research department via email or telephone. At this first contact, participants were informed about the study procedure, objectives, and insurance. Additionally, preliminary eligibility was ascertained. Criteria for patient in- and exclusion are summarized in Table 1. Participants of the CNSLBP group underwent screening by a clinician before participation in the study to screen for possible exclusion criteria. No minors were included. Participants of the control group had to be 18 years or older, without back pain (VAS = 0) and without disability caused by CNSLBP (Oswestry Disability Index = 0). No data was recorded before written informed consent was given by the participants.

**Table 1:** Criteria for inclusion and exclusion.

**Inclusion criteria**	• > 18 yrs. • Chronic non-specific low back pain
**Exclusion criteria**	• Pain in the neck or cervical spine that reduces active movement to less than 30° rotation on each side • Spinal pathology or surgery, major surgery during the previous six month • Whiplash during the last year • Known vestibular pathologies • Clinical signs of neurological damage • Traumatic injury to the musculoskeletal system during the previous six month • Inability to follow the procedures of the study, e.g. due to language problems, psychological disorders, dementia, etc. of the participant.

This study was carried out in accordance to the study protocol and with principles enunciated in the current version of the Declaration of Helsinki, the guidelines of Good Clinical Practice (GCP) issued by ICH, and the Swiss Law and Swiss regulatory authority’s requirements. The project was submitted to and accepted by the Ethics Committee for Northwest/Central Switzerland (EKNZ, EC number: 2014–337).

### Measurement protocol

All patient data were collected at one measurement event. Socio-demographic data like age, gender, and BMI were recorded prior to the measurements. Participants were given verbal explanation about the purpose and procedure of the study. Consent was given in written form by all participants. Participants of both groups were asked to report their low back pain level by using a visual analogue scale (VAS) for pain [43] scaled from “No pain” to “The worst pain imaginable”. Neck pain or active range of motion of the cervical spine were not assessed. The Oswestry Disability Index was used to assess the impact of pain on the patient’s disability in daily living. The Oswestry Disability Index has excellent test-retest reliability (r = 0.97) and internal consistency (Cronbach’s α: 0.90) [44, 45].

### Experimental setup

**Fig 2:**
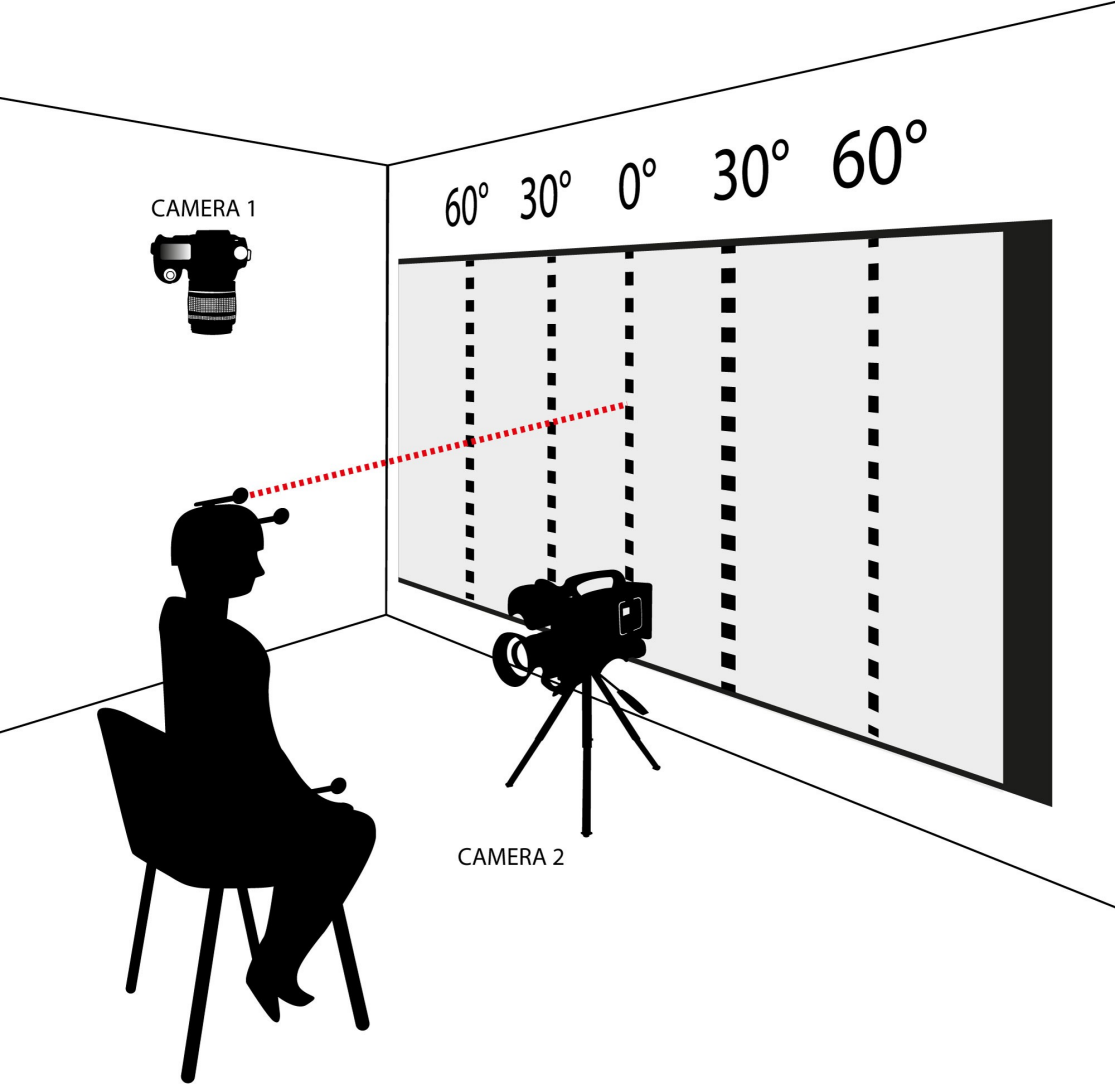
Measurement installation. A participant, blindfolded, with a computer mouse in the dominant hand, sitting on a chair with a laser pointer (1) fixed on a custom-made helmet. Vertical lines represent 0°, 30° and 60° angle to the left and the right side. One reflective marker (2) fixed frontally on the helmet, two reference markers (3) installed on the chair’s arm rests. Cameras installed both above (Camera 1) and in front of (Camera 2) the participant.

In preparation for the test, participants were seated on a chair and instructed not to move their shoulder blades away from the back of the chair. They were blindfolded to ensure visual deprivation during testing. A laser pointer was fixed on a custom-made helmet. Vertical lines on the wall facing the chair served as visual feedback for the assessor and were used to verbally instruct the participants to reach the starting position. The centre of the rotational axis of the angles was in the middle of the rear legs of the chair (Fig 2). Participants held a computer mouse in their dominant hand to react to the stimuli, a click on the left button indicated when they felt they had reached the neutral or the target position. One reflective marker was fixed on the front of the helmet, two reference markers were installed on the chair’s arm rests. Cameras were installed above and in front of the participant to record marker data with the motion tracking tool Templo v. 8.3 (Contemplas, Kempten, Germany). The frontal camera served for post measurement inspection to ensure only the head was rotated without trunk movement. The setup was pilot-tested in order to define optimal settings for the recording (e.g. light, camera distance, marker-positioning). The head was not repositioned by the researcher during performance of the five consecutive trials nor were verbal corrections given to achieve the starting position.

### Experimental procedure

One physiotherapist and two research assistants performed the measurements. Participants were given standardized word-by-word instructions prior to and during the tests following a test protocol, and were granted short rests between trials. Replication of neutral head position was tested first. The four subsequent positions were tested in randomized order. Participants were told to fulfil movements in the speed most comfortable to them within comfortable limits. Five consecutive repetitions per position were assessed.

#### Joint repositioning error of neutral head position

Measurement set up and procedures were adopted from Revel et al. [24]. Participants were instructed to sit comfortably and find their personal neutral position looking straight ahead. After holding their perceived normal position for 15s, they performed a full right and left rotation of the head and returned to neutral position for five times. Each neutral position was confirmed with a click on the mouse.

#### Joint repositioning error of 30° and 60° left and right rotation

The procedure of 30° and 60° target positions was based on Loudon et al. [42] and Nagai et al. [46]. Participants were verbally led to the correct position confirmed by the laser pointer meeting the vertical lines on the wall. They held this target position for 15s, returned to neutral position and replicated the target position for five times. Each target position was confirmed with a click on the mouse.

#### Data recording and processing

In the current study, the position measured was the position of the reflective marker on the head relative to the fixed markers on the arm rests of the chair. To record positional data of the marker, videos were tracked with Templo v. 8.3 (Contemplas, Kempten, Germany). Color and brightness were reduced in all video data to improve marker tracking accuracy. The frontal and horizontal two-dimensional marker positions were exported to MatlabTM version R2014b (Mathworks Inc., Natick, MA, USA) for further processing and syncing with reference data recorded from the mouse clicks. A custom-written algorithm identified marker-positions recorded with frontal and horizontal camera at the time-point of each click and produced three dimensional deviation data in degrees [°] for group-wise comparison: the absolute error relative to each individually set target position. The setup posed potential risk for recording errors. These may be caused by technical failure (recording software crashes) or human error (movements could not be performed). To mitigate these risks, the setting was tested prior to every measurement and patients were only included if the range of motion was not limited to less than 30° on each side. If data was lost or unattainable despite these measures, missing data was excluded list-wise for group comparisons.

### Statistical analyses

Statistical analyses were conducted with IBM SPSS Statistics (Version 23). Descriptive data was compared via appropriate analysis (chi-square, Mann-Whitney U-Test, t-test). The primary outcome was the mean deviation of the cervical JRE. The absolute error of the cervical JRE in degrees [°] of both groups in different positions was compared via Mann-Whitney U-Test for independent samples. Descriptive statistics, median and interquartile ranges with lower (25% quartile) and upper (75% quartile) limits were calculated. The significance level of the comparisons of cervical JRE was set at p = 0.01 (Bonferroni Correction for multiple comparisons with 0.05/5 = 0.01). In order to investigate the association between the repositioning accuracy and age Spearman correlation coefficients was calculated between the JRE for all five positions and age in addition, as the main outcome. The absolute error disregards the direction of over- or under-prediction, the over- and undershooting tendencies were determined to describe the performance of the head repositioning task in more detail. Over- and undershoots were counted based on Trelevean et al. (2003) [47] and a chi-square test was used for comparison of the two groups for each position for the executed shooting. The significance level of these comparisons was set at 0.05. Effect sizes *r* were calculated by dividing the z-value by the root of N (number of observations), with small (r = 0.2), medium (r = 0.5), and large (r = 0.8) effects [48].

## Results

### Participant characteristics

Forty-six participants with CNSLBP (n = 24, 54 ±16yrs standard deviation (SD), 14 females) and healthy individuals (n = 22, 36 ± 13yrs SD, 13 females) were included in the study. Demographic characteristics and pain values are reported in Table 2.

**Table 2:** Socio-demographic data of participants.

Groups	N	Gender (F:M)	Age (yrs.)	BMI (kg/m^2^)	VAS (cm)	ODI (%)	Activity per week (hrs.)
**Control**	22	14:8	39 ± 13	23.45 (± 2.6)	0	0	3.62 ± 2.6
**CNSLBP**	24	13:11	54 ± 16	24.54 (± 3.3)	3.04 ± 2.24	20.33 ± 9.5	3.26 ± 4.4
**Total**	46	27:19					
**p-value**		0.52*	0.01**	0.22***	0.01	0.01	0.01

N: Number of participants, BMI: Body Mass Index, VAS: Visual Analogue Scale of pain, ODI: Oswestry Disability Index; Activity per week = Activity per week was all regular active exercise, e.g. walking, biking, gym, yoga etc.;

* = chi-square test;

** = Mann-Whitney U-Test;

*** = t-test.

### Joint repositioning error

**Fig 3:**
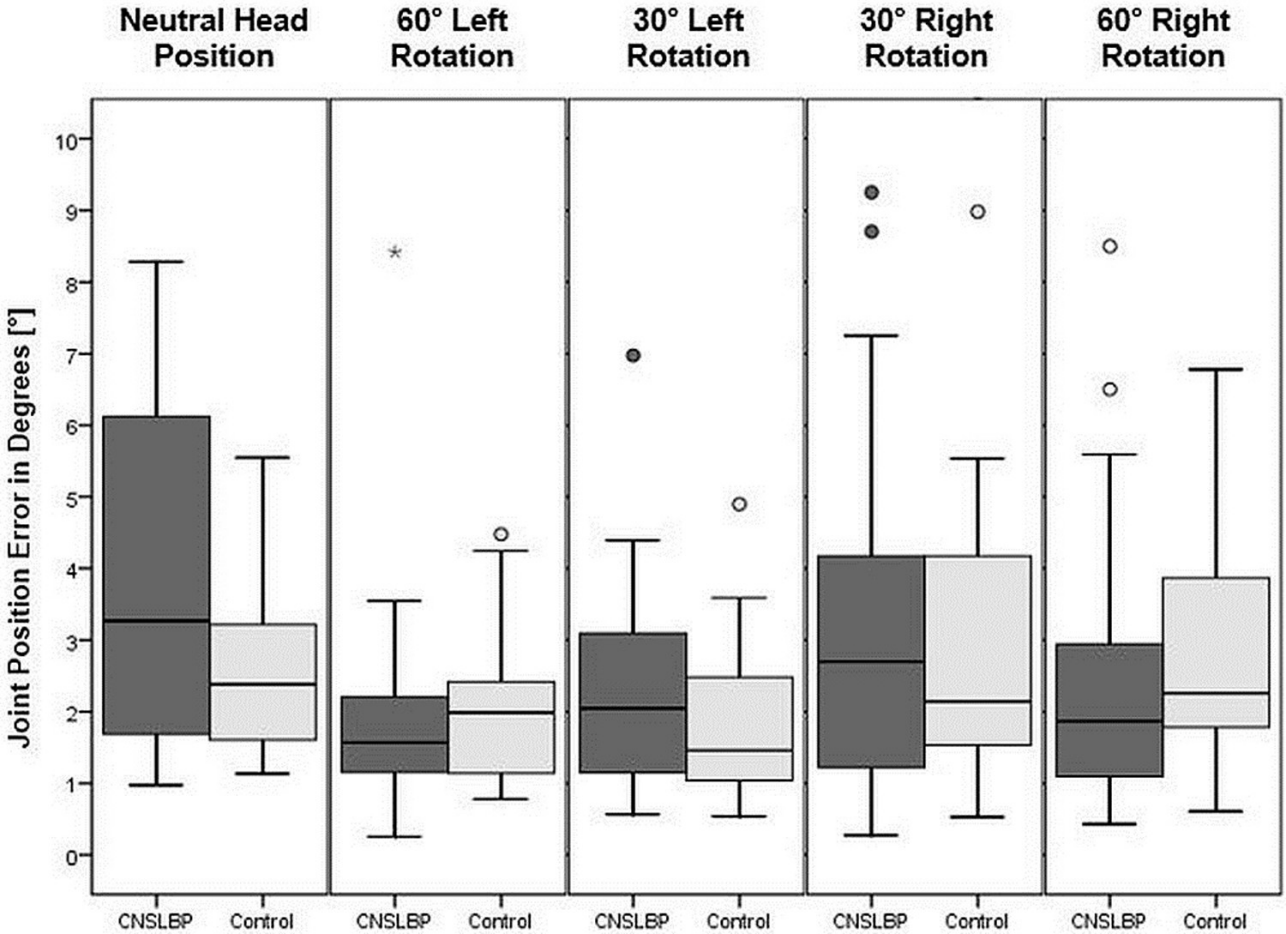
Boxplots of position data of the 5 different positions. Boxplots of the medians and Interquartile Range of the JRE of five different positions. Outliers are expressed as * and dots.

**Fig 4:**
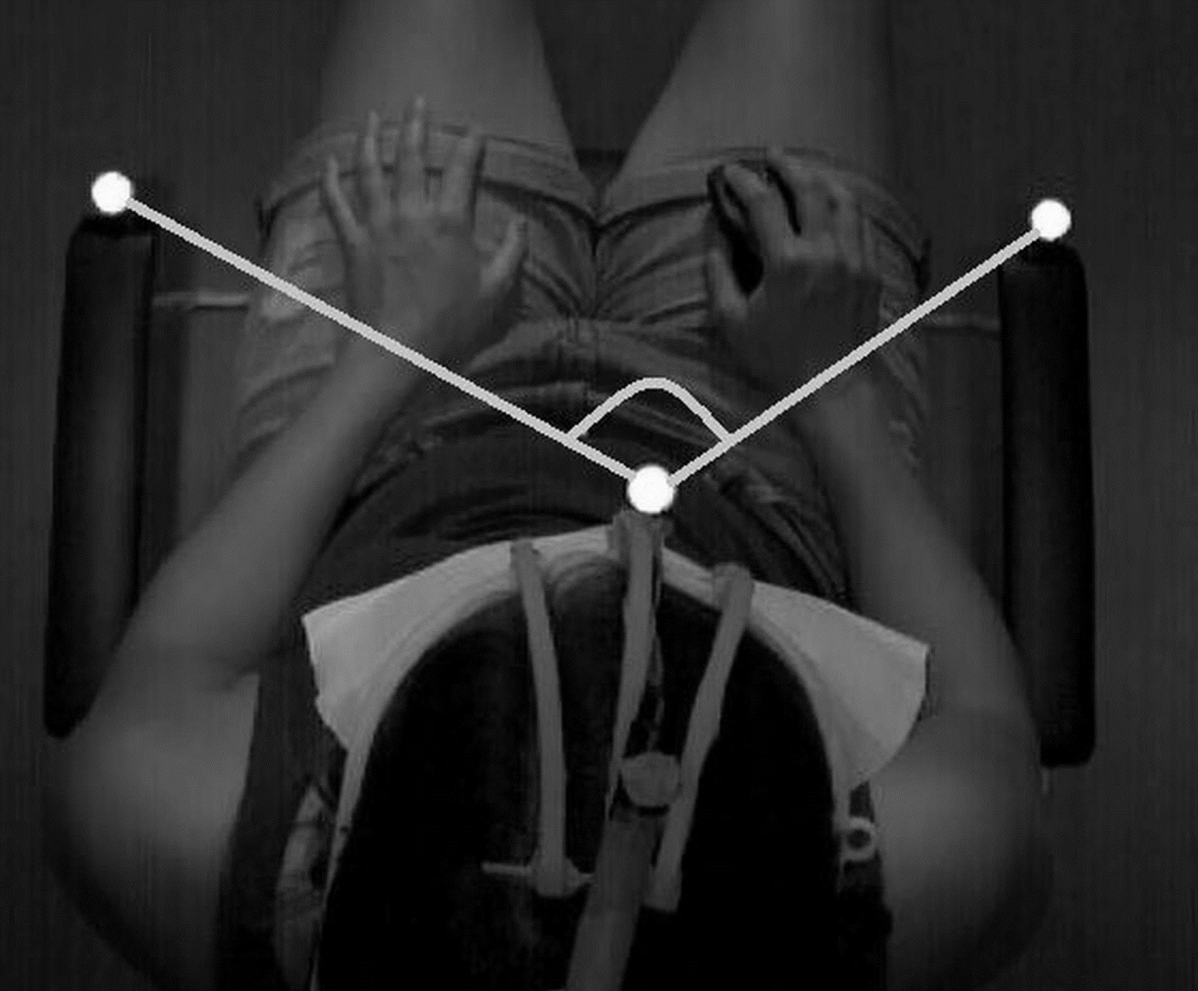
Angle between reflective markers. *(1)* Marker on left arm rest. *(2)* Marker on head gear. *(3)* Marker on right arm rest. *Gray line*: 3-dimensional angle between markers in horizontal perspective. The angle between markers changed during head-movement with head marker.

**Fig 5:**
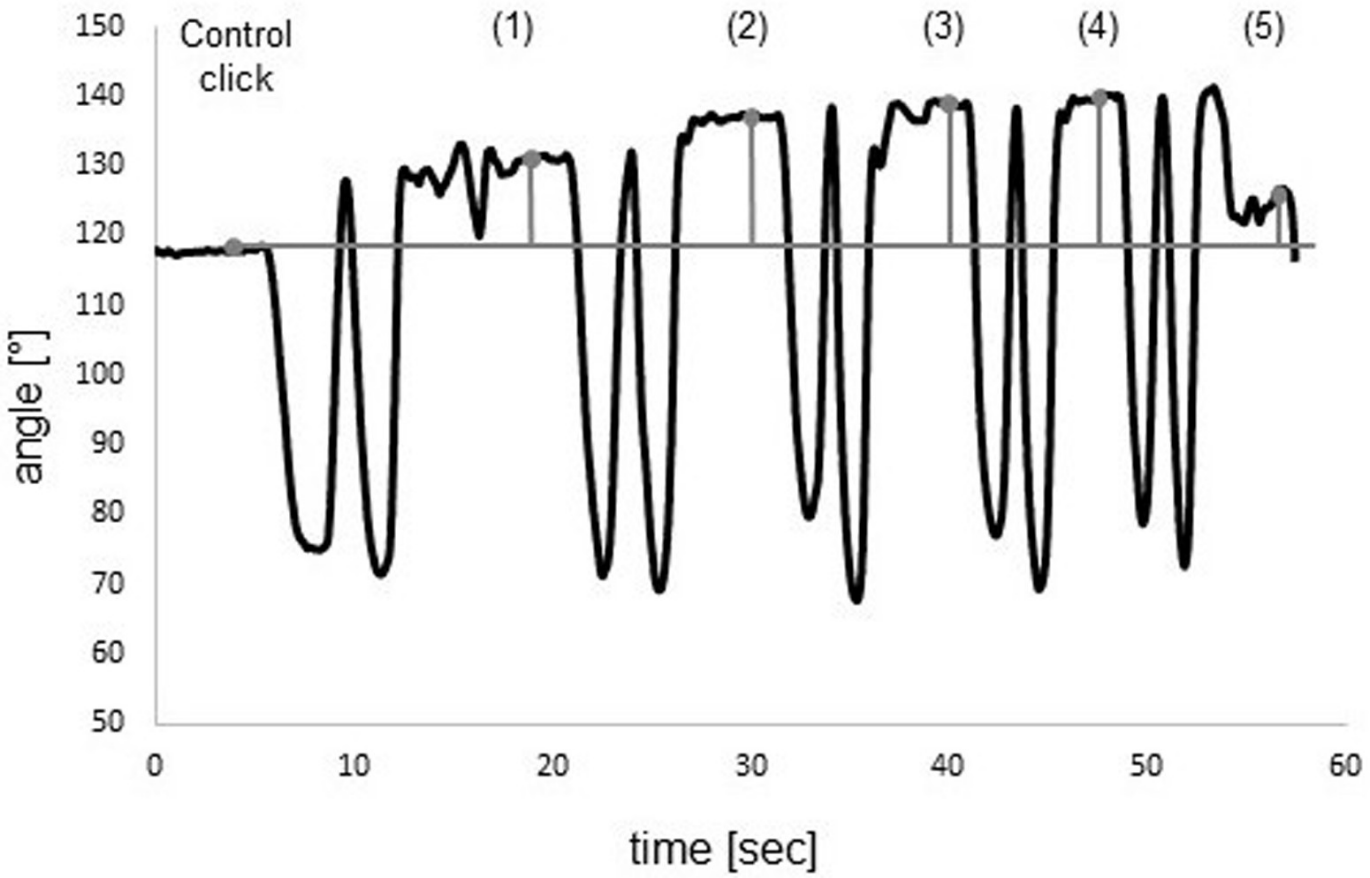
Large deviation in neutral head repositioning. *Black line*: Row angle between head and chair markers during measurement. *Gray line*: Control click and five consecutive clicks to confirm position. Deviation from black line to grey line represents the deviation of the row between-marker angle to the control angle. Mean deviation (absolute error): 8.1°.

**Fig 6:**
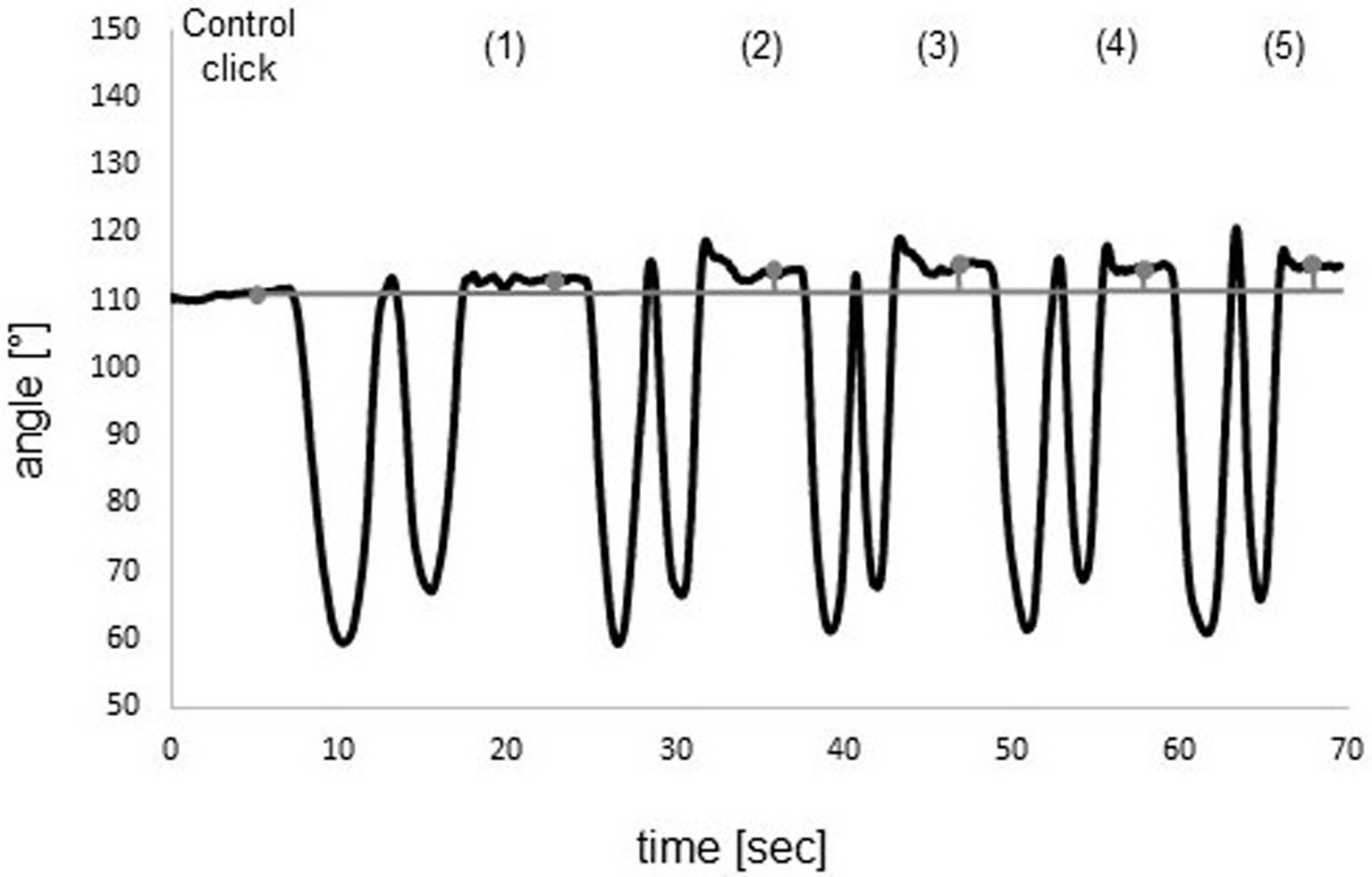
Small deviation in neutral head repositioning. *Black line*: Row angle between head and chair markers during measurement. *Gray line*: Control click and five consecutive clicks to confirm position. Deviation from black line to grey line represents the deviation of the row between-marker angle to the control angle. Mean deviation (absolute error): 1.3°.

The mean absolute errors of cervical JRE relative to individually set targets, measured in degrees, between the target and the reproduced position in the five testing trials were calculated for all participants in the two groups. All medians of the mean repositioning errors and interquartile ranges with the results of Mann-Whitney U-Tests comparing the two groups are shown in Table 3. Boxplots of all five positions are shown in Fig 3. For description of between-marker angle refer to Fig 4. In Figs 5 and 6, curves of the row between-marker angle of a large and a small deviation from target angle performed by two participants can be seen.

**Table 3:** Results of cervical joint repositioning error.

Group	N	CJRE [°] Median	IQR	p-value*/ES	CJRE [°] Mean	SD
**Neutral Head Position**						
CNSLBP	22	3.27	1.65–6.27	0.21/0.24	3.94	2.59
Control	20	2.38	1.58–3.22		2.58	1.16
**60° Left Rotation**						
CNSLBP	21	1.57	1.04–2.25	0.36/0.16	1.95	1.68
Control	22	1.99	1.13–2.54		2.08	1.05
**30° Left Rotation**						
CNSLBP	23	2.05	1.15–3.13	0.31/0.12	2.41	1.57
Control	21	1.46	1.03–2.48		1.89	1.13
**30° Right Rotation**						
CNSLBP	23	2.70	1.12–4.70	0.98/0.03	3.25	2.53
Control	22	2.14	1.51–4.19		3.19	2.58
**60° Right Rotation**						
CNSLBP	22	1.87	1.09–3.24	0.23/0.18	2.57	2.10
Control	22	2.26	1.71–4.07		2.96	1.82

N = Number of participants, CJRE = Cervical Joint Repositioning Error; IQR = Interquartile Range;

* = Mann-Whitney U-Test;

ES = effect size; SD = Standard Deviation.

#### Correlation between age and JRE

Spearman correlations showed no association between age and cervical JRE for both groups, where for neutral head position r = 0.009, 60° left r = 0.152, 60° right r = 0.031, 30° left r = -0.056 and 30° right rotation r = -0.018.

#### Characteristics of repositioning

An undershoot tendency was found for 60° left rotation in the CNSLBP group with a statistically significant group difference (p<0.01). Most positions were more often undershot by both groups, except neutral head position with a slightly more often overshoot by both groups. Descriptive statistics of under- and overshooting is presented in Table 4.

**Table 4:** Under- and overshooting.

	CNSLBP		Control			
	N	Group %^1^	N	Group %^1^	Total%^2^	p-value*
**Neutral Head Position**						
Undershooting	**10**	45.5	**6**	30.0	38.1	0.30
Overshooting	**12**	54.5	**16**	70.0	61.9	
**60° Left Rotation**						
Undershooting	**19**	90.5	**12**	54.5	72.1	0.01
Overshooting	**2**	9.5	**10**	45.5	27.9	
**30° Left Rotation**						
Undershooting	**14**	60.9	**12**	57.1	59.1	0.80
Overshooting	**9**	39.1	**9**	42.9	40.9	
**30° Right Rotation**						
Undershooting	**16**	66.7	**10**	47.6	57.8	0.20
Overshooting	**8**	33.3	**11**	52.4	42.2	
**60° Right Rotation**						
Undershooting	**14**	63.3	**17**	77.3	70.5	0.32
Overshooting	**8**	36.4	**5**	22.7	29.5	

N = number of participants,

* = Pearson Chi-Square group comparison;

^1^ = % of distribution in group;

^2^ = total % of over-/undershooting.

## Discussion

The purpose of this study was to compare neck proprioception in patients with CNSLBP and in healthy individuals by measuring the cervical JRE. The main finding was no identifiable differences between CNSLBP patients and healthy controls in measures of neck proprioception.

In the current study, no statistically significant group-difference was found between patients with CNSLBP and healthy individuals for cervical JRE. The results show, however, slight group-differences in accuracy and variability in repositioning of neutral head position, as patients with CNSLBP showed a greater JRE and a greater inter quartile range (IQR) when repositioning to the neutral head position. When repositioning to 60° and 30° left and 30° right rotations, both groups achieved approximately the same results in JRE and IQR. In 60°, there was a tendency towards a greater JRE and greater IQR in the control group. Effect sizes of all positions were small, with 0.24 for neutral head position as the highest effect size.

There are different measures to quantify the deviation from the target position. According to Rausch Osthoff, 2015 [14], the JRE can be expressed as an absolute error, constant error, or variable error. The absolute error represents the error magnitude and is the unsigned difference between the starting and finish position. It indicates the accuracy of the deviation from the starting position [14, 20, 25]. CE represents the error magnitude direction such that constant error indicates bias toward a particular direction, therefore a negative constant error typically represents a bias in the undershooting direction. Variable error describes the variability of the participants’ performance equivalent to the standard deviation of RE. High Variable error values reflect high variability in repositioning. As absolute error has often been assessed in the neck and lumbar spine repositioning, it was decided to use it in this study to enable comparison with existing works where neck and lumbar repositioning were investigated.

Cervical repositioning error was used to indirectly measure proprioception. Although it is not possible to measure actual sensory information transferred from receptors to the CNS with this measurement, it is currently the primary measure in people with chronic neck pain with the underlying assumption that poor performance in this test reflects abnormal neck proprioception, including afferent input [10, 11, 14, 21, 37, 49]. Absolute errors for horizontal movements of Revel et al. [24] were 3.5° (±0.82°) for healthy participants and 6.11° (±1.59°) for neck pain patients (p<0.01).

This study was implemented to better understand the nature and consequence of somatosensory impairment for the better understanding of spinal control problems in back pain. There are possible explanations, why the present study could not identify differences in neck proprioception of patients with low back pain. In the present study, participants of the CNSLBP group were 15 years older on average compared to the control group. This, together with levels of physical activity of the individuals in the different groups, poses to be a potential biasing factor [50, 51]. The literature on the relationship between age and repositioning error is, however, conflicting, suggesting that there may be other factors beyond age that may also influence repositioning sense [38]. Findings from Artz et al. [52] support our results, as they neither found mean repositioning errors that correlated with age. Only Lansarde et al. [53] reported age-related declines in position sense in people aged over 60 years [53]. Other studies suggest a reduction of cervical range of motion at an older age [53, 54]; Youdas et al. [55] described a loss of 7° per decade in the horizontal plane.

The influence of the vestibular system on proprioception, particularly the speed of motion, has been shown to play a role [37]. The faster the head moves, the more the JRE represents vestibular afferent rather than cervical afferent information (cut-off 2.1°/s) [37]. In our study, participants were told to move in the way most comfortable to them. However, we did not measure the speed of motion and, thus, this may have varied between participants. Therefore, a conclusion about influence of the vestibular system on the measurement cannot be made.

To avoid interference and new input during each measurement, participants were not allowed to open their eyes. Additionally, participants were not touched to re-center their heads to the prior starting position. In Treleaven et al. [47], the examiner manually repositioned the participant’s head back to the original starting position before each trial and the participants were able to visually re-center their starting position prior to each new movement direction. This procedure may lead to a different deviation of target positions as errors and deviations may be larger for overshooting or smaller for undershooting when returning the head exactly to the target position during measurement.

In a systematic review, de Vries et al. [37] suggested that the repositioning error was generally higher in the neck pain group than in the control group when measured over 6 trials or more. In this study, five consecutive trials were conducted. It is possible that six or more trials could reduce the vulnerability to outliers in the statistic. Furthermore, to avoid learning effect, no replication of a trial in case of failure was possible. Artz et al. [52] reported lower repositioning errors when adopting upright compared to flexed postures in healthy individuals. In the current study, all participants were seated on the same chair to assure a standardized procedure.

It was attempted to avoid fatigue or decreased attention by the short duration of about 10 minutes per participant for the entire JRE measurement. General and muscular fatigue were not assessed but could possibly have been higher during evening hours. Furthermore, the allocation of 30° and 60° left and right rotations were conducted in a random order to control for potential sequence effects.

The findings of this study may suggest that sensorimotor control is affected on other levels of the central nervous system. Current research explains the idea that chronic pain is not a concept with solely abnormalities in afferent sensory inputs and spinal cord reorganization, but a brain network disorder with a reorganization of sensorimotor cortical regions [56]. Mano et al. [56] found a consensus pattern of modular reorganization involving extensive, bilateral regions of sensorimotor cortex, with sensorimotor cortex nodes being less inclined to form pairwise modular links with other brain nodes. In addition, Apkarian et al. [57] described that with the possibility of brain imaging studies, an active role of the cortex in processing pain is assumed and the prior assumption of a rather passive role of the cortex seems complemented. Future research should find methods to further identify the localization and source of these aberrations within the central nervous system.

## Conclusion

In summary, the ability to accurately position the head does not seem to be impaired in patients with CNSLBP. The results of the comparison of mean cervical JRE between CNSLBP patients and healthy controls showed no statistically significant group difference in any of the head positions. The results show, however, slight group-differences in accuracy and variability in repositioning of neutral head position, as patients with CNSLBP showed a greater JRE and a greater inter quartile range when repositioning to the neutral head position. These findings may suggest that sensorimotor control is affected on other levels of the central nervous system. Current research describes brain network disorders and cortical changes in low back pain. Future research should find methods to identify the exact localization and source of these aberrations.

## Supporting information

S1 Table Missing data. Missing data occurred because of three different failures: video, patient or tracking failure.
(TIF)
