# Supplementary figures and images for: Comparison of proprioceptive acuity of the cervical spine in healthy adults and adults with chronic non-specific low back pain: A cross-sectional study
Source: PLoS One. 2019 Jan 10;14(1):e0209818. doi: 10.1371/journal.pone.0209818 (PMC6328243; doi:10.1371/journal.pone.0209818)

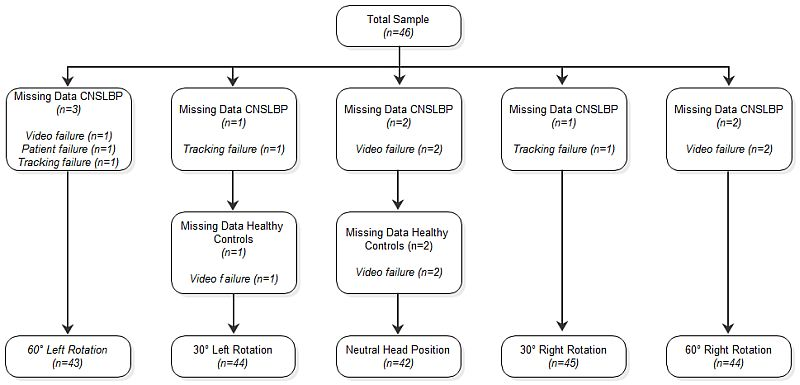

Supplement: S1 Table — Missing data occurred because of three different failures: video, patient or tracking failure. (TIF) [file pone.0209818.s001.tif]
